# Clinical and Epidemiological Characterization of Acute Chagas Disease in Casanare, Eastern Colombia, 2012–2020

**DOI:** 10.3389/fmed.2021.681635

**Published:** 2021-07-23

**Authors:** Claudia Yaneth Rincón-Acevedo, Andrea Stella Parada-García, Mario Javier Olivera, Fernando Torres-Torres, Liliana Patricia Zuleta-Dueñas, Carolina Hernández, Juan David Ramírez

**Affiliations:** ^1^Centro de Investigaciones en Microbiología y Biotecnología-UR, Facultad de Ciencias Naturales, Universidad del Rosario, Bogotá, Colombia; ^2^Maestría en Salud Pública, Escuela de Medicina y Ciencias de la Salud, Universidad del Rosario, Bogotá, Colombia; ^3^Grupo de Parasitología, Instituto Nacional de Salud, Bogotá, Colombia; ^4^Secretaría de Salud de Casanare, Yopal, Colombia

**Keywords:** *Trypanosoma cruzi*, acute Chagas disease, DTU, outbreaks, Casanare, Colombia

## Abstract

**Background:** Chagas disease (CD), caused by the protozoan *Trypanosoma cruzi*, is considered a public health problem in Latin America. In Colombia, it affects more than 437,000 inhabitants, mainly in Casanare, an endemic region with eco-epidemiological characteristics that favor its transmission. The objective of this study was to describe the clinical and epidemiological characteristics of the cases of acute CD in Casanare, eastern Colombia, in the period 2012–2020.

**Methods:** In the present study, 103 medical records of confirmed cases of acute CD were reviewed. The departmental/national incidence and fatality were compared by year; the climatological data of mean temperature, relative humidity, and precipitation per year were reviewed and plotted at IDEAM (Colombian Meteorology Institute) concerning the number of cases of acute CD per month, and it was compared with the frequency of triatomines collected in infested houses by community surveillance. Univariate, bivariate, and multivariate analyses were performed, comparing symptoms and signs according to transmission routes, complications, and age groups.

**Results:** The incidence was 3.16 cases per 100,000 inhabitants, and the fatality rate was 20% in the study period. The most frequent symptoms included: fever 98.1%, myalgia 62.1%, arthralgia 60.2%, and headache 49.5%. There were significant differences in the frequency of myalgia, abdominal pain, and periorbital edema in oral transmission. The main complications were pericardial effusion, myocarditis, and heart failure in the group over 18 years of age. In Casanare, TcI Discrete Typing Unit (DTU) has mainly been identified in humans, triatomines, and reservoirs such as opossums and dogs and TcBat in bats. An increase in the number of acute CD cases was evidenced in March, a period when precipitation increases due to the beginning of the rainy season.

**Conclusions:** The results corroborate the symptomatic heterogeneity of the acute phase of CD, which delays treatment, triggering possible clinical complications. In endemic regions, clinical suspicion, diagnostic capacity, detection, and surveillance programs should be strengthened, including intersectoral public health policies for their prevention and control.

## Background

Chagas disease (CD), caused by the parasite *Trypanosoma cruzi*, continues to be a significant cause of morbidity, disability, and mortality, mainly in Latin America. It is estimated that it affects 6–7 million people globally, constituting an emerging threat for several non-endemic countries (1, 2). The disease has an acute phase and a chronic phase characterized by an unpredictable clinical course, ranging from the absence of symptoms to severe diseases with cardiovascular, gastrointestinal, or neurological involvement (3). *Trypanosoma cruzi* presents high genetic variability given that six Discrete Typing Units (DTUs) have been identified that are distributed throughout the American continent (TcI-TcVI) and an additional DTU associated mainly with anthropogenic bats called TcBat described in Brazil, Colombia, Panama, and Ecuador (4, 5). Additionally, genetic variability has been detected in TcI DTU, finding parasites associated with sylvatic transmission cycles and a genotype mainly associated with domestic transmission TcI_Dom_ (6). Consequently, the clinical forms and the severity of the manifestations are diverse between regions and individuals, considering the host's immune response and a plethora of *T. cruzi* virulence factors (7).

Clinically, the acute phase of CD can be asymptomatic or present non-specific symptoms that include prolonged fever and general malaise, estimating that only 1–2% of cases in this phase are identified (4, 8); mortality can range between 10 and 80%, which is widely variable depending on the diagnosis and timely treatment (2). Additionally, in the severe form of the acute phase, complications such as myocarditis, heart failure, arrhythmias, atrioventricular blocks, and to a lesser extent, meningoencephalitis may occur, situations that increase mortality (9). When the disease is not diagnosed or treated in the acute phase, the patient evolves into the chronic phase, with the risk of developing irreversible chronic Chagas heart disease characterized by complex ventricular and atrial arrhythmias, bradyarrhythmias, atrioventricular block, apical aneurysm, ventricular dysfunction, and heart failure (10–12) and digestive complications such as megaesophagus and megacolon (13).

Among the various transmission routes of the disease, the vector and oral routes stand out, the latter associated with acute outbreaks with a reported lethality for Latin America between 1 and 35% (14, 15). In some countries of the Southern Cone, such as Brazil, Venezuela, Bolivia, Ecuador, Peru, Argentina, and French Guyana (16), cases of acute CD due to vector transmission and oral outbreaks have been described due to contamination of food with feces of wild triatomines or with secretions from reservoirs. Colombia has also been an important scene of acute Chagas outbreaks, which had increased since 1992 when the first outbreak was identified. The departments of Norte de Santander, Magdalena, Santander, Putumayo, Arauca, Cesar, Antioquia, Chocó, Atlántico and Casanare have reported various outbreaks and isolated cases (17). Despite the high number of oral transmission outbreaks, there are failures in the clinical care of patients given the non-specificity of the symptoms, the clinical and epidemiological ignorance of the disease, which translates into low suspicion of it and affects the presentation of complications and fatality (4).

In Colombia, it is estimated that more than 437,000 infected inhabitants and around 5 million are at risk of acquiring CD (18). The department of Casanare in the Colombian Orinoquia is endemic for CD and has ecoepidemiological characteristics that favor the transmission of the disease. According to the National Institute of Health (INS) of Colombia, in 2017 40.3% of chronic cases and 20% of acute cases in the country were identified in Casanare. The situation in 2018 was similar to 2017, while in 2019, 50.9% of acute CD cases in the country came from Casanare (19). This department has reported outbreaks of oral transmission with the lethality of up to 50% and outbreaks associated with occupational exposure with a significant number of cases (20, 21). Currently, Colombia has a program to interrupt the domiciliary vector transmission of *T. cruzi* by *Rhodnius prolixus*, which means that oral transmission may increase and, with this, the presence of acute cases (22, 23).

In Casanare, ten triatomine species (Hemiptera: Reduviidae) have been reported, where *R. prolixus* and *Triatoma maculata* stand out, the first with presence in 17 of the 19 municipalities (24, 25). Likewise, abundant populations of sylvatic *R. prolixus* have been identified associated with native palms *Attalea butyracea* and palms introduced in agro-industrial crops such as *Elaeis guineensis* with high rates of natural infection by *T. cruzi* (24). A high presence of *R. prolixus* infected with *T. cruzi* has been shown in homes in rural areas, which increases in the low rainfall season and decreases in the months of higher rainfall (26, 27). On the other hand, Casanare is the department of the country with the highest number of acute CD, both isolated cases and outbreaks; between 2012 and 2020, 8 outbreaks of presumed oral transmission were reported, and acute cases have been reported in 52.6% (10/19) of the municipalities.

The insufficient knowledge of the epidemiology and clinical presentation of the disease has been derived from case reports and small series of cases. These are the product of different investigations but are not part of the routine epidemiological surveillance of the event. To close the knowledge gap on this little-known and devastating infection, we present an extensive series of cases of acute infections by *T. cruzi* confirmed by the laboratory in the National Public Health Surveillance System in the period 2012–2020, from one of the most endemic departments of Colombia. The results of this study will permit plan health services, guide decision-makers, developing health policies that improve detection and increase awareness of this disease.

## Methods

### Ethics Approval

This project was approved by the ethics committee of Universidad del Rosario Act number 426 from Jul 30, 2020.

### Methodological Design

A retrospective, observational and descriptive case series study was carried out from the review of medical records of patients from Casanare notified to the Public Health Surveillance System - SIVIGILA- with acute CD in 2012–2020. The National Public Health Surveillance System (SIVIGILA), regulated in Colombia in 2006, is responsible for the objective, systematic and constant observation and analysis process of health events, supporting the orientation, planning, execution, monitoring, and evaluation of public health practice. SIVIGILA has the Primary Data Generating Units (UPGD), which correspond to Health Service Provider Institutions (IPS) in which cases of the various diseases (including CD) object of public health surveillance are detected and reported (28). A systematic literature review was carried out to verify circulating DTUs in the department of Casanare. Additionally, the database of vectors obtained from community surveillance was further obtained.

### Study Area

The department of Casanare is located in the Colombian Orinoquía, with 381,554 inhabitants (National Administrative Department of Statistics, DANE 2019) distributed in 19 municipalities, of which 75.2% of the population resides in the municipal capitals. The annual average temperature varies from 6 to 27°C. Extremes of drought and humidity characterize the climate during the year; there is a rainy period with abundant rainfall between April to November and a dry or summer period from December to March; relative humidity is between 60 and 90%, and it has three types of landscapes that range from 100 meters above sea level to 3,800 meters above sea level. Ninety-five percentage of the department has a warm, humid climate on the foothills of the eastern mountain range and the flat zone (29). Its landscape is characterized by extensive savannas of grasslands, shrubs, industrial crops of rice and African palm, as well as patches of palm forests on the banks of rivers, streams, wetlands, and lagoons. 57% of the department comprises flooded savannas where numerous rivers of the Orinoco River basin flow. The most important economic activities are cattle ranching, agriculture, and mining (30); most rural dwellings are immersed in landscape matrices whose main structural component are wild palms. This area provides biological, ecological, and environmental conditions, which favor the ecoepidemiological cycles of the parasite *T. cruzi* (27).

According to the 2005 DANE estimate, 13.36% of the population lives in inadequate housing, 5.61% with inadequate services, 17.36% in critical overcrowding, and there is a housing deficit of 47,3%, this figure refers to homes with housing shortages both due to qualitative and quantitative deficits (29).

## Information Sources

### Human Patients

The Department Health Secretariat was requested to provide a database of reported cases for the study period and 100% of the medical records that were part of the epidemiological field investigations. The transmission route classification was established following field epidemiological investigations and following the surveillance protocol for Chagas disease in Colombia, including the epidemiological, entomological, and laboratory components (sampling).

The data were used to build a database in Microsoft Excel 365 software with sociodemographic, clinical, laboratory, and epidemiological characteristics of each of the cases (see Supplementary Table 1). The municipality constructed summary tables of acute cases from 2012 to 2020, which were supplemented with demographic data from the national statistical service (DANE) according to the report “Estimation and projection of total national, departmental and municipal population 1985–2020” (31) and the gross incidence was calculated dividing the number of cases by the projected population for each year and the lethality for Colombia and Casanare. These data were graphed with R studio.

To plot the number of cases of acute CD by the municipality, four quartiles were established, grouping the total of cases in the period 2012–2020, and the map was made in QGIS version 3.10.11 La Coruña. For the definition of the case, the definitions established in the SIVIGILA were used (32).

### Vectors and Reservoirs

The database of triatomines collected by community surveillance in the 2012–2020 period was used. Through the community triatomine surveillance strategy, the population captures entomological material that are subsequently taken to the departmental entomology laboratory for classification and diagnosis of *T. cruzi* infection (33). From this database, the information on the main triatomine species collected that infest houses were analyzed. It was extracted by the municipality and by month of collection. Likewise, the literature about the triatomines collected in Casanare was reviewed (25).

For the climatological variables, the data of the 1981–2020 climatological average of the variables precipitation, average temperature, and relative humidity for Casanare were consulted at the Institute of Hydrology, Meteorology and Environmental Studies (IDEAM), which were analyzed for each month together with the average number of triatomines collected by community surveillance monthly. Lastly, reservoir information compiled from the epidemiological surveillance of acute CD outbreaks was included.

### DTUs Circulating in Casanare Department

Finally, a literature review was carried out in the Pubmed, Google Scholar databases, and the BVS Regional Portal, with the terms, *Trypanosoma cruzi*, Discrete Typing Unit, DTU, Casanare or Colombia, humans; obtaining 12 articles that described the circulating DTUs by the municipality and for the department in general (see Supplementary Table 2). The information from the previous articles was extracted, discriminating it by type of DTU, species (reservoirs, vectors, and humans), and municipality. Later, it was graphed on a map using QGIS version 3.10.11 La Coruña.

### Statistical Analyses

The demographic and clinical characteristics of the cases were analyzed. A database was designed in Microsoft Excel (Microsoft, Redmond, USA) that contained all the study variables, and the data analyzes were performed with Epi Info 7.2.2 and Stata (release 14, Stata Corporation, College Station, TX, USES). The descriptive and univariate analysis included demographic and clinical variables. Continuous variables were summarized using measures of central tendency and dispersion. The categorical variables were presented as frequencies and percentages. The t-student, Chi-square, or Fisher test was used to determine the significance of the difference in the bivariate analyses: comparison of signs and symptoms according to transmission route, complications according to transmission route, age groups, and differences according to outbreaks and isolated cases. For the analysis of complications, the main complications associated with acute CD in the literature were categorized: pericardial effusion, myocarditis, heart failure, arrhythmia, pericarditis, cardiac tamponade, meningoencephalitis, and atrial fibrillation in the group of under-aged patients (pediatric age) compared to the group over 18 years (adults) (34, 35). Multivariate analyzes were performed using logistic regression models. Odds Ratios (OR) and their corresponding 95% confidence intervals (95% CI) were calculated for each variable. In all cases, a value of *p* < 0.05 was considered statistically significant. The results are presented in tables, graphs, and maps.

## Results

### Number of CD Cases Reported, 2012–2020

**Figure 1:**
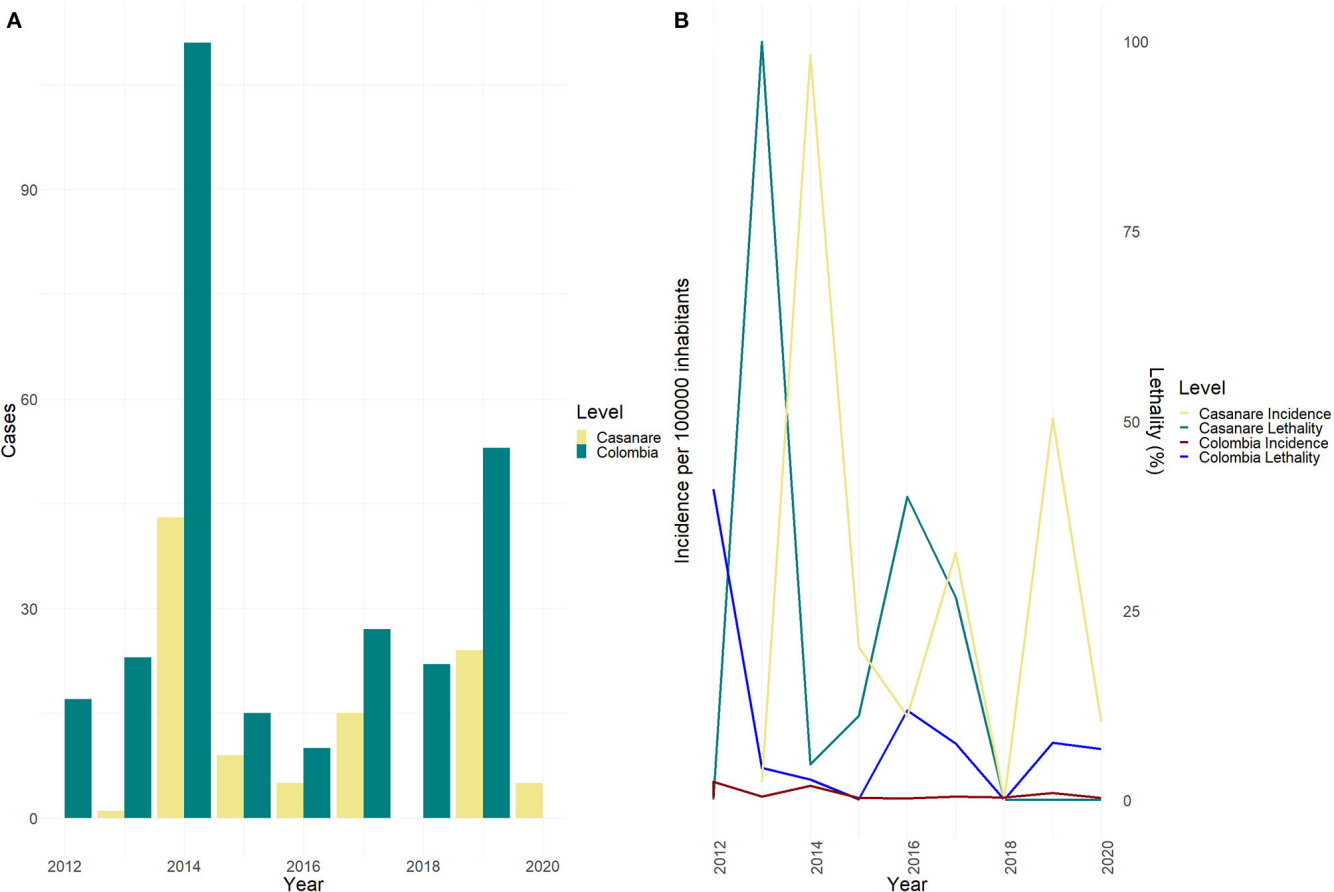
Cases and incidence of Chagas disease by year. **(A)** Acute Chagas disease Cases by Year, Colombia vs. Casanare 2012–2020. **(B)** Incidence per 100,000 inhabitants and fatality (%) due to acute Chagas disease, Colombia vs. Casanare 2012–2020.

Between 2012 and 2020 in Colombia, 293 laboratory-confirmed cases of acute CD were notified to the Public Health Surveillance System (SIVIGILA). In Casanare, 232 cases were reported, 34.4% (*n* = 80) were discarded for not meeting the case definition, 12.5% (29/232) children of mothers positive for *T. cruzi* (probable cases of congenital transmission), 8.6% (20/232) repeated records of people who were notified in different health institutions. For the analysis of the present study, 103 cases were included: 83.5% (*n* = 86) confirmed by laboratory, 12.6% (*n* = 13) probable cases that shared characteristics of time, place, and person with some outbreaks, and 3.8% (*n* = 4) confirmed by epidemiological link; the latter correspond to patients who died without laboratory confirmation, during an outbreak in which other cases were confirmed. 35.2% (103) of the acute CD cases reported in Colombia between 2012 and 2020 were from Casanare, with an average of 11 (±14) cases per year (Range 0–43). The general incidence for Casanare was 3.16 cases per 100,000 inhabitants, and the general lethality was 20%, considerably exceeding the national incidence and lethality during the study period (Figure 1).

### Sociodemographic Characteristics

**Figure 2:**
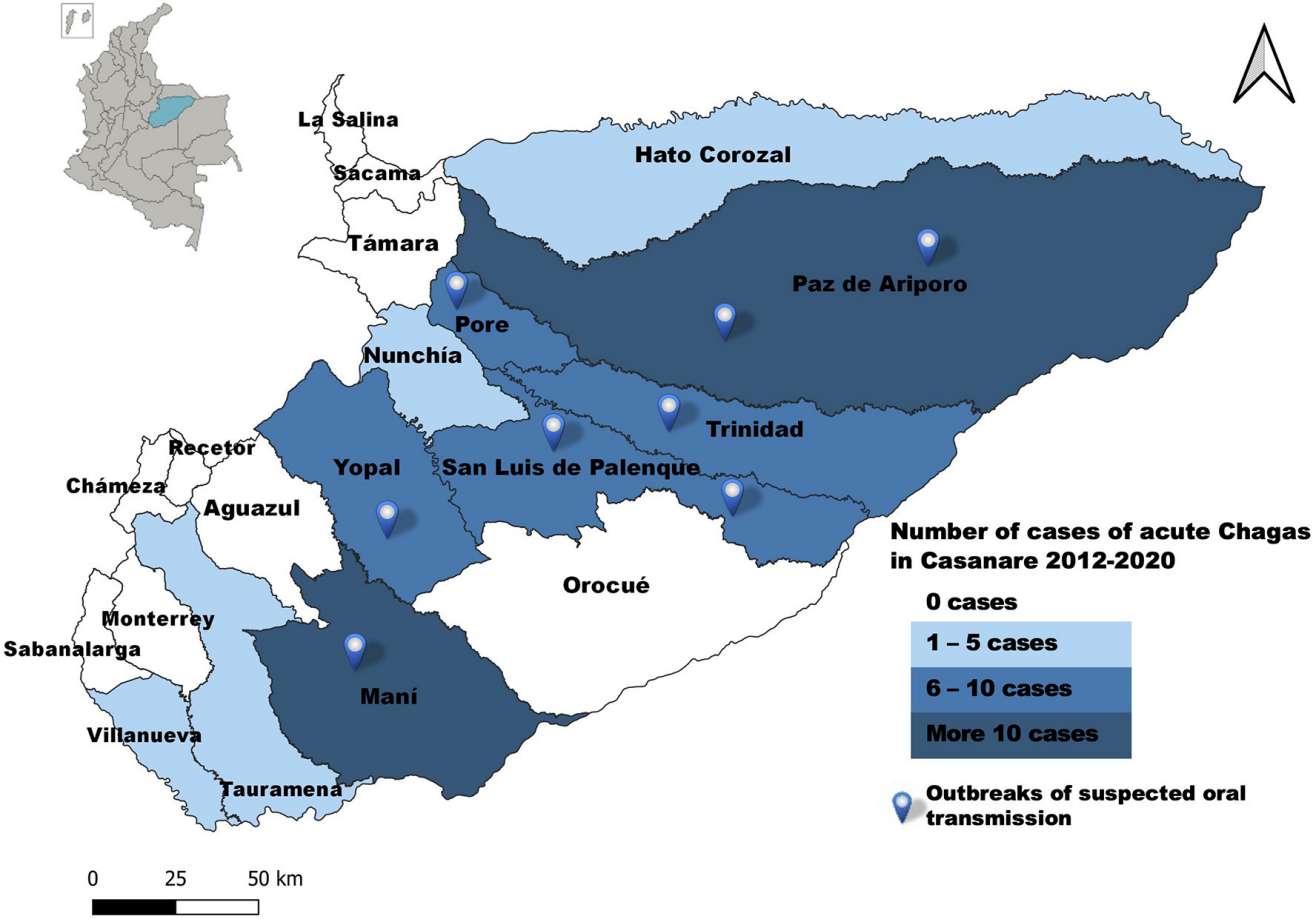
Geographic distribution, acute Chagas disease by municipality of origin, Casanare 2012–2020.

69.9% (*n* = 72) of the cases occurred in males, 96.1% (*n* = 99) came from rural areas, 57.3% (*n* = 59) with a contributory affiliation regime (the contributory regime is a set of rules that govern the connection of individuals and families to the General System of Social Security in Health) (36). The average age was 33 years (±15) (Range from 0 to 78 years) and a higher percentage of cases in the group aged 30–44 years (see Supplementary Table 3). Cases were reported from 52.6% (*n* = 10) of the municipalities of Casanare; Paz de Ariporo and Maní have reported the highest number of cases. Outbreaks of presumed oral transmission occurred in Paz de Ariporo, Pore, Trinidad, San Luis de Palenque, Maní, and Yopal (Figure 2).

### Clinical Presentation

98.1% of the subjects presented febrile syndrome with a mean temperature of 38°C (±1.3°C), sudden onset, prolonged, followed by other non-specific symptoms such as myalgias (62.1%) and arthralgias (60.2%). The entry signs of the parasite, such as the Romaña sign and inoculation chagoma, were rarely identified. People with acute CD consulted on day 17 (±9.5) after the onset of symptoms (Range: 0–45 days) and, on average, had a medical consultation before diagnosis with a maximum of 6 previous consultations.

Signs/symptoms were compared according to transmission route, and there were statistically significant differences in myalgia (*p* = 0.012), abdominal pain (*p* = 0), periorbital edema (*p* = 0.001) in oral transmission. In contrast, the romaña sign occurred with a higher proportion in vector transmission with a statistically significant difference (*p* = 0.0007) (Table 1). Statistically significant differences were identified between arthralgias (*p* = 0.012), abdominal pain (*p* = 0.00), periorbital edema (*p* = 0.001) in cases of oral transmission compared with the vector route. The differences between the other signs and symptoms were not statistically significant (*p* > 0.05).

**Table 1:** Frequency of acute CD signs/symptoms, according to transmission route, Casanare 2012–2020.

**Signs/symptoms**	**Oral transmission (*****n*** **=** **85)**		**Vector transmission (*****n*** **=** **18)**		***P*-value**
	**Frequency**	**%**	**Frequency**	**%**	
Fever	84	98.8	17	94.4	0.777
Myalgia	58	68.2	6	33.3	**0.012**
Arthralgia	55	64.7	7	38.9	0.077
Headache	41	48.2	10	55.6	0.76
Tachycardia	34	40.0	10	55.6	0.342
Dyspnoea	34	40.0	5	27.8	0.481
Abdominal pain	39	45.9	0	0.0	**0**
Chest pain	22	25.9	7	38.9	0.408
Tachypnea	21	24.7	4	22.2	1
Asthenia	21	24.7	4	22.2	1
Chill	21	24.7	3	16.7	0.554
Adinamia	19	22.4	4	22.2	1
Facial edema	18	21.2	4	22.2	1
Sickness	18	21.2	3	16.7	1
Hepatomegaly	16	18.8	4	22.2	0.747
Periorbital edema	9	10.6	8	44.4	**0.001**
Vomit	16	18.8	1	5.6	0.294
Cough	15	17.6	2	11.1	0.731
Palpitations	12	14.1	1	5.6	0.456
Diarrhea	10	11.8	2	11.1	1
Lower limb edema	9	10.6	3	16.7	0.435
Romagna sign	3	3.5	6	33.3	**0.0007**
Skin rash	7	8.2	0	0.0	0.349
Hepatosplenomegaly	7	8.2	0	0.0	0.349
Bradycardia	4	4.7	0	0.0	1
Splenomegaly	4	4.7	0	0.0	1
Chagoma	1	1.2	2	11.1	0.078
Retroocular pain	2	2.4	1	5.6	0.441

*Bold values are statistically significant*.

### Diagnosis

Regarding the diagnostic images, 60.2% of the patients had a chest X-ray, 54.4% an electrocardiogram, 33% an echocardiogram, and 27.2% an abdominal ultrasound; only 1.9% had Holter. The main finding on the chest X-ray was cardiomegaly, on the electrocardiogram sinus tachycardia and right bundle branch block; the echocardiogram found pericardial effusion more frequently, while the abdominal ultrasound mainly identified hepatomegaly (Table 2).

**Table 2:** Main findings reported in diagnostic images, Acute CD, Casanare 2012–2020.

**Main changes in diagnostic images**	**Frequency**	**%**
**X-ray**		
Abnormal	28	45.2
Cardiomegaly	18	64.3
Pleural effusion	8	28.6
**Electrocardiogram**		
Abnormal electrocardiogram	29	51.8
Sinus tachycardia	12	41.4
Right bundle branch block	8	27.6
Nonspecific repolarization disorder	8	27.6
AV block	4	13.8
ST supran level	3	10.3
Left bundle branch block	2	6.9
Atrial fibrillation	2	6.9
T wave inversion	2	6.9
Sinus bradycardia	2	6.9
Ventricular extrasystoles	1	3.4
**Echocardiogram**		
Abnormal echocardiogram	24	85.7
Pericardial effusion	21	87.5
Cardiac tamponade	2	8.3
**Abdominal ultrasound**		
Abnormal abdominal ultrasound	28	100.0
Hepatomegaly	20	71.4
Splenomegaly	4	14.3
Free liquid in cavity	4	14.3

The most frequently used diagnostic tests for confirming cases of acute CD were the thick smear (56.3%) and the micromethod (15.5%). 23.3% of the cases had positive qPCR. The parasite load of 12 patients who tested positive by qPCR was obtained, and the median load was 21.6 (parasite equivalents/mL). 71.8% of the patients underwent serological tests, mainly ELISA or IFAT, and two seroconverted from negative to positive, classified as acute infection (see Supplementary Table 4). The main hematological alterations found were: anemia 22.3% (*n* = 23), leukocytosis 17.4% (*n* = 18), macroplatelets 14.5% (*n* = 15), thrombocytopenia 13.5% (*n* = 14) and leukopenia 7.7% (*n* = 8). 68.9% (*n* = 71) of the cases had an increase in aspartate aminotransferase, 50.5% (*n* = 52) in alanine aminotransferase and 48.5% (*n* = 50) increased creatinine.

### Differential Diagnosis

The primary differential diagnoses of admission included dengue (23.3%), leptospirosis (6.8%), malaria (4.9%), and brucellosis (3.9%). 79.6% of the cases were hospitalized, with an average hospital stay of 7 (±8) days (Range 0–35 days). There were no statistically significant differences between hospitalization according to the vector or oral route of transmission (*p*-value: 0.758) or hospitalization differentiated between outbreaks and isolated cases (*p*-value: 0.745).

### Complications

34.9% (*n* = 36) cases presented at least one complication, mainly pericardial effusion, myocarditis, and heart failure. There were no statistically significant differences between the presence of complications and the route of transmission (*p*-value > 0.05). Complications occurred more frequently in the group over 18 years old (84.5%). The differences with the group under 18 years old were not statistically significant (*p*-value > 0.05). The highest frequency of complications occurred in cases associated with outbreaks (76.4%) than isolated cases; these differences were not statistically significant (Table 3).

**Table 3:** Type of complications according to transmission route, Acute Chagas disease, Casanare 2012–2020.

**Complications**	**No. cases**	**%**	** <18 years**		**≥18 years**		***p*-value**	**Oral transmission**		**Vector transmission**		***p*-value**	**Outbreaks**		**Isolates cases**		***p*-value**
			**No. cases**	**%**	**No. cases**	**%**		**No. cases**	**%**	**No. cases**	**%**		**No. cases**	**%**	**No. cases**	**%**	
Pericardial effusion	21	56.8	2	9.5	19	90.5	0.514	18	85.7	3	14.3	1	17	81.0	4	19.0	1
Myocarditis	18	48.6	6	33.3	12	66.7	0.052	14	66.7	4	19.0	0.513	13	61.9	5	23.8	0.509
Heart failure	14	37.8	1	7.1	13	92.9	0.69	13	61.9	1	4.8	0.454	12	57.1	2	9.5	1
Cardiac arrhythmia	9	24.3	1	11.1	8	88.9	1	6	28.6	3	14.3	0.189	6	28.6	3	14.3	0.371
Pericarditis	4	10.8	0	0	4	100	1	4	19.0	0	0.0	1	3	14.3	1	4.8	1
Cardiac tamponade	2	5.4	0	0	2	100	1	2	9.5	0	0.0	1	1	4.8	1	4.8	0.352
Meningoencephalitis	1	2.7	1	100	0	0	0.55	1	4.8	1	4.8	0.32	1	4.8	1	4.8	0.352
Atrial fibrillation/atrial flutter	2	5.4	0	0	2	100	1	2	9.5	0	0.0	1	2	9.5	0	0.0	1

### Treatment

In 90 cases (87.3%), etiological treatment was administered, and the most frequently used drug was Benznidazole (72.8%). The information provided for the study did not report possible adverse effects associated with the drugs or the need to change drugs. The multivariate analysis did not show associations between mortality and clinical variables (all with *p* > 0.05).

### Lethality

Ten of the cases under study had a fatal outcome; of these, 50% were men, with an average age of 26 years (±21), minimum age of 7 months, and a maximum of 64 years, 30% of the deceased cases were younger than 5 years. Eighty percentage of the deaths occurred in outbreaks of oral transmission. The overall fatality for the department was 10%, and in some outbreaks of oral transmission, the fatality reached 50%. Subjects died on average 20 (±21) days after the date of symptom onset (Range: 3–71 days). Of the total number of deaths, eight autopsies were performed; the main findings reported in the autopsies were the presence of amastigotes of *T. cruzi* in the myocardium and severe acute inflammatory processes; additionally, three autopsies reported encephalitis with the presence of amastigotes of *T. cruzi*. In two deaths, the previous findings were not presented, and deaths due to acute CD were considered by sharing characteristics of time, place, and person with confirmed cases in the framework of an outbreak. On the other hand, a case of reactivation was presented in a 64-year-old patient with HIV infection, with sudden onset of neurological symptoms, loss of sphincter control, cerebral toxoplasmosis, and *T. cruzi* encephalitis was diagnosed. There were no statistically significant differences between death according to the vector or oral route of transmission (*p* = 0.685).

### Triatomine and Mammal Species Identified Per Municipality

**Figure 3:**
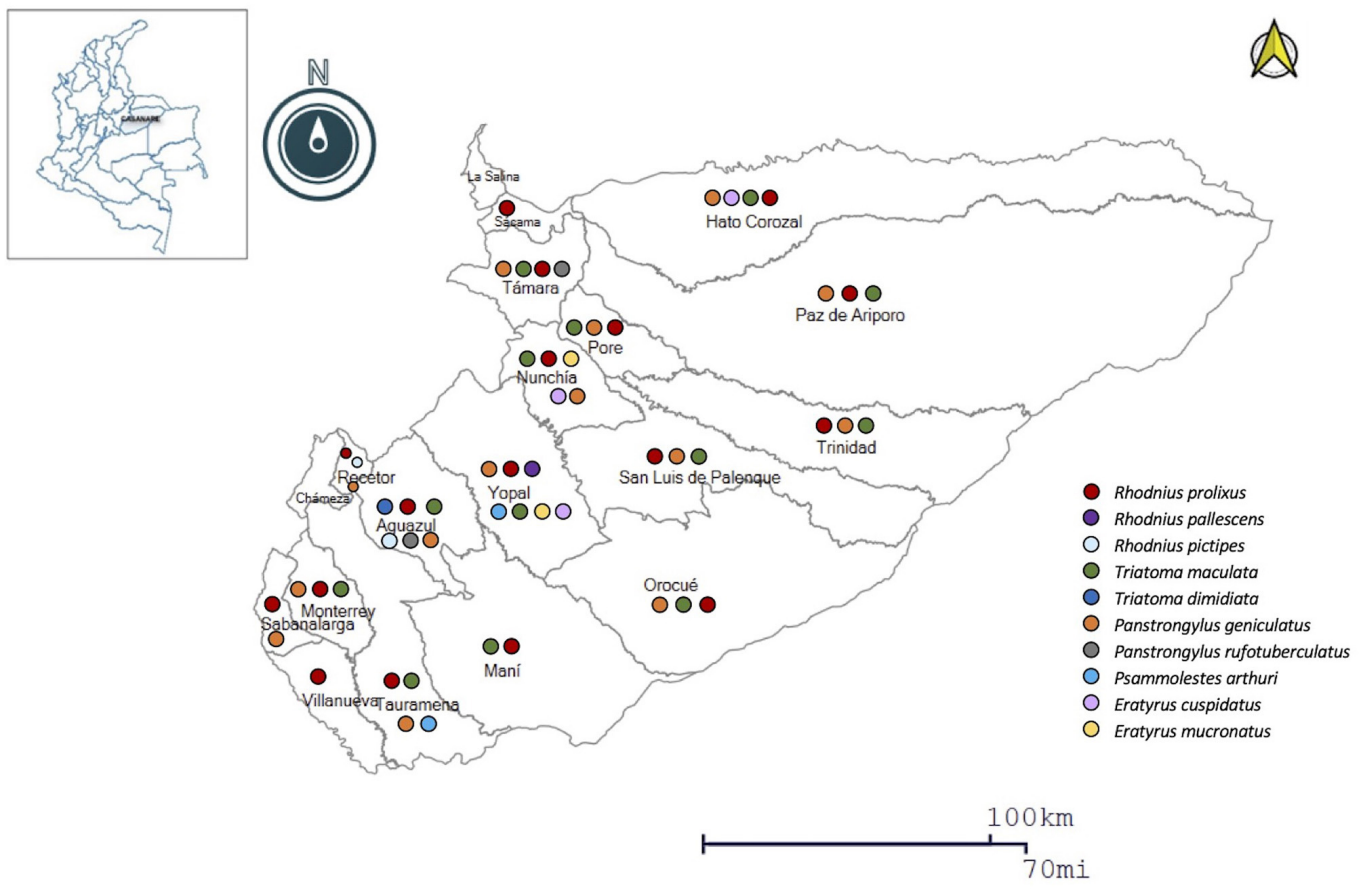
Geographic distribution of the main triatomine species in Casanare.

According to the information obtained through the community surveillance strategy in the 2015–2020 period. Ten species of triatomines captured by the community in the intra-domicile have been identified, among which *R. prolixus* stand out in 89.5% (17/19) of the municipalities, *P. geniculatus* 73.5% (14/19) and *T. maculata* in 68.4% (13/19) (Figure 3).

Regarding the distribution of *T. cruzi* reservoirs in Colombia, the highest prevalence in mammals of four taxonomic orders has been reported: Didelphimorphia (35.0%); Carnivora (17.0%); Rodentia (6.0%); and Chiroptera (15.0%); identifying in Casanare positive reservoirs of the taxonomic orders: Artiodactyla, Carnívora, Chiroptera, Pilosa, and Didelphimorphia (37).

### Discrete Typification Units Identified in Casanare

**Figure 4:**
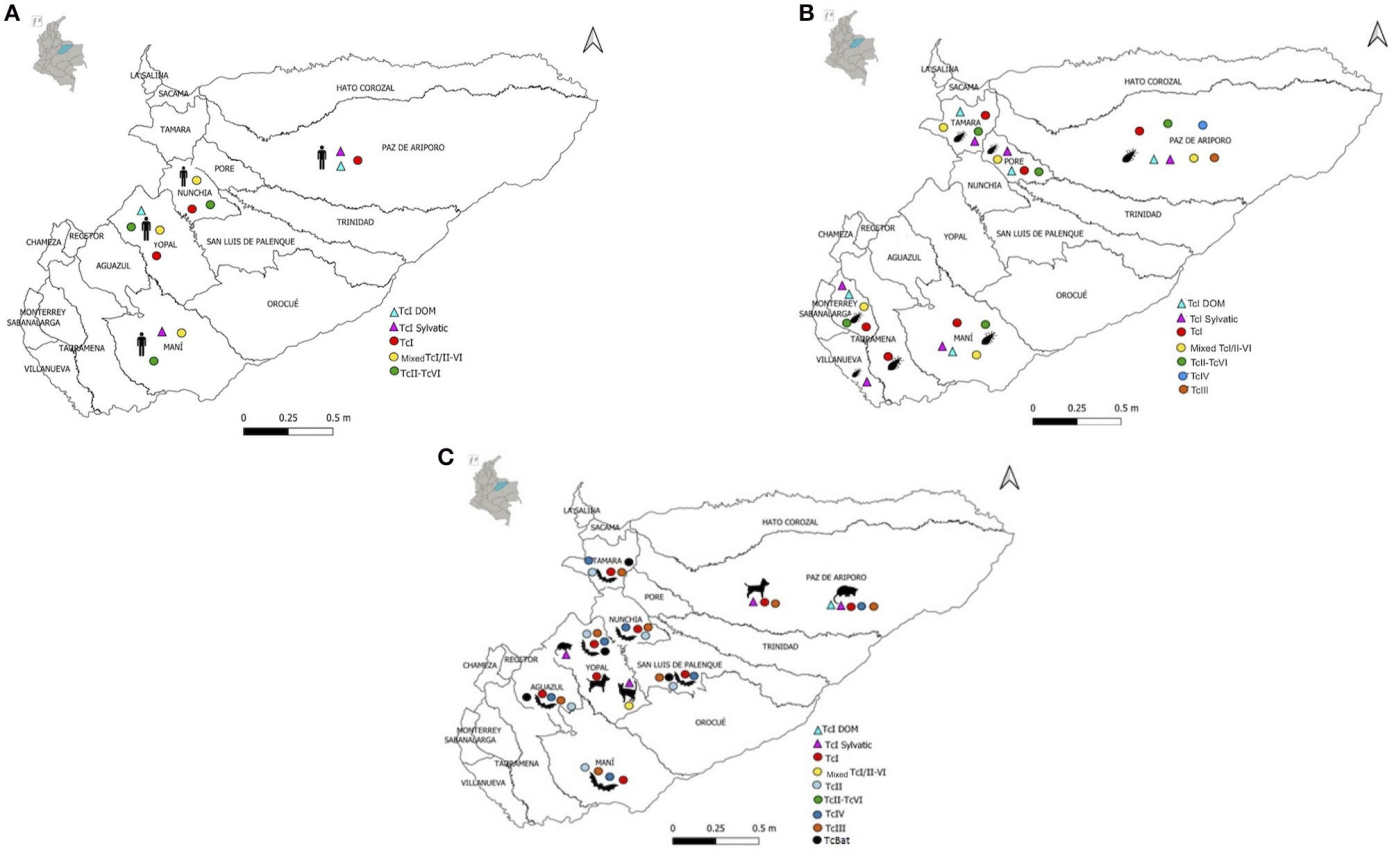
Geographic distribution of DTUs identified in different Casanare species; **(A)** Distribution of DTUs in humans (*Homo sapiens*); **(B)** Distribution of DTUs in triatomines; **(C)** Distribution of DTUs in reservoirs (*Canis familiaris, Didelphis marsupialis, Felis catus*).

The detection of all DTUs described for *T. cruzi* throughout the epidemiological circuit has been achieved. In triatomines, TcI_Dom_, TcI_Sylvatic_, and mixed TcI/TcII-VI infections have been found mostly; in reservoirs TcI_Dom_, TcI_Sylvatic_, and TcIII; in humans TcI_Dom_, TcI_Sylvatic_, and mixed TcI/TcII-VI infections. In Figure 4, the geographical distribution of DTUs in the different municipalities of the department can be observed (Figure 4).

Studies carried out in triatomines have mainly identified the DTUs TcI, TcII, TcIII, TcIV, and TcVI in *R. prolixus, T. maculata, P. geniculatus*, and *Psammolestes arthuri* (27, 38–41). In a study carried out in children with chronic Chagas disease from this region, DTU TcI was identified more frequently (42). In Casanare, the presence of the TcBat genotype was described for the first time in Colombia (43), TcIII was identified in the nine-banded armadillo (*Dasypus novemcinctus*) (44), and the presence of TcI_Sylvatic_ has been described in other wild species such as rats (*Rattus rattus*), bush rats (*Proechimys* sp.), howler monkeys (*Alouatta* sp.) and anteater (*Tamandua tetradactyla*) (45).

### Weather Conditions

**Figure 5:**
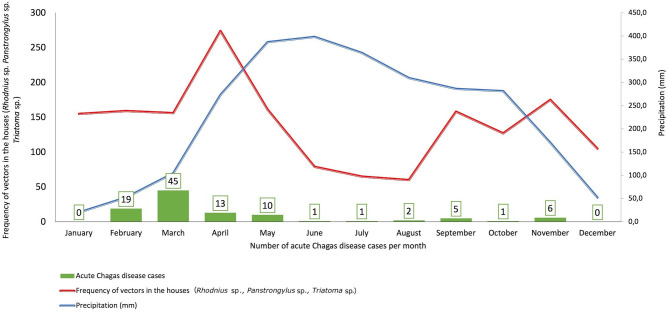
Climatic conditions, frequency of vectors by community surveillance vs. Number of Acute Chagas disease cases, Casanare 2012–2020.

According to the weather conditions reported by IDEAM, Casanare's average rainfall in January was 20.2 mm and starting this month, it begins to increase, being 273.1 mm in April, May with 387 mm and the maximum average in June with 398.1 mm. When comparing with the results of the community surveillance of triatomines, an increase in the frequency of collection of vectors was found inside the houses in April and May, which coincides with the more significant precipitation and the greater number of cases of acute CD reported (Figure 5). In the acute orally transmitted CD outbreaks in 2014, 2016, and 2020, a decrease in precipitation was observed below average. In the 2014 outbreak, which has been the largest to date in Colombia, these outbreaks were related to reservoirs, mainly *Didelphis marsupialis*, which due to climatic conditions, approached human dwellings in search of food and water (21, 46, 47).

## Discussion

In this study, some epidemiological patterns can be discerned. However, it has been described in the literature that most patients with acute manifestations of CD are children (48). In the present investigation, the highest percentage of cases occurred in young adults (between 30 and 34 years old), a behavior similar to that reported in Brazil in the most extensive series of cases documented to date; however, in Casanare, the most significant affectation occurred in males, which could be related to economic activity, in contrast with Brazil (49) (Supplementary Table 3). The most frequent clinical presentation was a febrile syndrome, with sudden and prolonged onset, mainly accompanied by myalgias, arthralgias, and headache (Table 1). Fever remains the main symptom, similar to that found in other regions of Colombia (50, 51) and even in countries like Brazil and Venezuela (52–54).

In the study period, the incubation average was 17 days (±9.5), higher than that reported in Mérida-Venezuela, where symptoms are described in an average of 12 days ± 3 (55). This finding has clinical and epidemiological relevance since it reaffirms the importance and need to suspect this pathology in people with prolonged febrile syndromes. In whom parasitological tests should be performed, and timely treatments can be established, the subjects under study had up to 6 medical consultations before diagnosis. It is noteworthy that, even though Casanare is considered an endemic region for CD, 23.3% of the study subjects had dengue as a diagnosis of admission, which delays treatment and leads to the onset of possible complications. This situation has also been described in other series of cases in Colombia (56, 57). Even in other countries where the diagnosis of acute CD is made post-mortem (58) by identifying amastigotes of *T. cruzi* in the myocardium. Infectious diseases such as malaria, leptospirosis, brucellosis, rickettsiosis, and histoplasmosis, among others, are part of the differential admission diagnoses identified in our series of cases and other countries (59). This reinforces the need to train medical personnel in endemic regions for a timely diagnosis of acute CD.

Regarding the signs such as the Romaña sign and inoculation chagoma, these were rarely identified in the series of cases analyzed (9 patients with Romaña sign and 3 with chagoma) and the described in Brazil where out of 233 cases, 6 presented front door signs (49). In contrast to the descriptions made by Dias in a series of cases from Brazil and Argentina where the frequency of these signs was between 12.5 and 95.5% (60). In a study carried out in Barinas-Venezuela, signs of entry of the parasite were found in 45.8% (27/59) of the cases (61). In general, gastrointestinal symptoms such as abdominal pain, nausea, vomiting, and diarrhea were found more frequently in cases associated with oral transmission in the present study, similar to what was identified in other outbreaks of oral transmission reported both in Colombia and in South America (43–45). Hepatomegaly and splenomegaly infrequently occurred, 19.5 and 3%, respectively, with an average age of 25 years, unlike the reports in Bambuí-Brazil where they were reported in 66 and 31%, respectively, being marked in children from zero to 2 years, in whom it was considered a marker of poor prognosis (60). In the analysis carried out in this study, 20% of the subjects with hepatomegaly died, which could also be considered an indicator of poor prognosis that leads to strengthening the clinical follow-up of acute CD cases.

The frequency of electrocardiographic anomalies in the present investigation was 51.8%, and the main alterations were sinus tachycardia, right bundle branch block, and non-specific repolarization disorder (Table 2), different from that reported by Pinto et al. in Brazil, where in 233 cases studied, 51.1% had EKG alterations with a predominance of diffuse alterations in ventricular repolarization, low voltage of the QRS fields and sinus tachycardia (49). In the study of an outbreak due to oral transmission in a school in Caracas-Venezuela, 61 patients were studied; in those under 18 years of age (48), T wave anomalies and ST abnormalities were more frequent and supraventricular arrhythmias predominated in the adults (62). This could be explained by the difference in the virulence of the *T. cruzi* strains and, additionally, by the parasite-host interaction that can produce different responses from the immune system and other organs (63).

34.9% of the subjects had at least one complication in the present series of cases, mainly pericardial effusion, myocarditis, and heart failure. Different results from those described in Santander-Colombia, where 12 cases were studied, 66.6% had heart failure as the main complication, followed by pericardial effusion with 41.6% (50). Some authors suggest that in cases of oral transmission, more significant complications could occur in adults than children since the former would consume a greater quantity of food containing a more significant number of infective metacyclic of *T. cruzi* (55, 64). In the present study, although complications occurred more frequently in those over 18 years of age, the difference with those under 18 was not statistically significant. Likewise, although it was found that the highest frequency of complications occurred in cases associated with outbreaks in which the oral transmission hypothesis was raised, the difference was not significant compared to vector transmission (Table 3). The results of this study corroborate the importance of timely detection and treatment of people in the acute phase of the disease and long-term follow-up of patients undergoing treatment. The need for other studies complemented with molecular epidemiology is raised to generate knowledge that allows the control of the disease, considering the epidemiological, ecological, and cultural characteristics of regions such as Casanare.

This study shows that mortality was higher in males, in the young adult population (26 years ± 21), a situation that can be related to work activity; 30% of subjects who died were from the group under 5 years old, findings different from those described in the series of cases in Brazil between 1988 and 2005 where fatality was more frequent in adults over 50 years old (49). However, both results shown in this study and those described in the region's countries reaffirm that there may be a greater risk of fatality in extreme ages. On average, the subjects died 20 days after symptoms onset (±21), and the main finding in the autopsies was the presence of amastigotes of *T. cruzi* in the myocardium, also reported in subjects who have died in Venezuela and Brazil (64–66). It is noteworthy that one of the cases analyzed corresponded to reactivation in an immunosuppressed patient with HIV, who presented neurological symptoms with a diagnosis of *T. cruzi* encephalitis. In this regard, the literature mentions that neurological manifestations in 75% accompany reactivations in patients with HIV-AIDS to 90% of cases (67, 68). In this context, Chagas disease control programs must include HIV screening in patients with chronic Chagas disease and vice versa, especially in endemic areas, where reactivation of Chagas disease should be considered in the differential diagnosis of meningoencephalitis among HIV-infected patients (69, 70).

The fatality due to acute Chagas disease was higher in Casanare than in Colombia in the study period. Several reasons could explain this: first, most of the cases analyzed were of oral transmission, which has been associated with higher fatality (48); likewise, in Casanare, a greater prevalence of sylvatic TcI DTU has been found, the genotype most likely associated with the oral transmission which has been identified in vectors, reservoirs, and humans, reaffirming the role of this DTU in the oral transmission of the disease (21), since the infection in humans is accidental, given the intrusion of the parasite into the domestic environment from the sylvatic cycle, as previously described in other outbreaks in Colombia (71), Venezuela, French Guyana and Brazil (17, 72). It is also important to bear in mind that, in remote regions of Colombia such as Casanare, timely access to the health system is limited. In Casanare, 65% of the health services with hospitalization are in the capital of the department with a proportion of 12 beds per 10,000 inhabitants (29), and 84.2% of the municipalities only have institutions that provide health services of the first level of care where the medical staff and supplies are insufficient, which could be related to the delays in the timely identification of patients with Chagas disease and increases the risk of complications.

On the other hand, the most frequently used diagnostic methods to demonstrate parasitemia in the study subjects were thick blood smear and concentration methods such as the micromethod with the positivity of 56.3 and 15.5%, respectively (Supplementary Table 4). In a study carried out in Venezuela, positive thick smear percentages of 34% were found, and xenodiagnosis was the second most frequent method, with a positivity of 61% (49). The 23.3% of the cases under study were diagnosed with qPCR; however, this test is not part of the diagnostic algorithm validated in Colombia, and although it has shown high sensitivity, it is usually available only in reference or research laboratories (59, 73). This demonstrates the need to perform qPCR at least in outbreak studies in Colombia, strengthening epidemiological surveillance, especially in endemic regions.

Regarding the genetic diversity of *T. cruzi*, in Casanare, as in Colombia, TcI predominates in domestic and sylvatic cycles of transmission, and in patients in the chronic phase, coinfection of TcI-TcII, TcI-TcIV, as well as the unique presence of TcII (74). A study conducted in children with chronic Chagas disease from Casanare identified the TcII-TcVI genotypes (42). On the other hand, in Casanare, ten species have been identified, of which *R. prolixus* stands out in 89.5% of the municipalities, and the TcI-TcVI DTUs have been identified in *R. prolixus, T. maculata, P. geniculatus, and Ps. arthuri* captured in the department (27, 38–40) (Figures 3, 4).

The appearance of acute CD cases in Casanare is due to human interaction with the domestic and sylvatic cycles circulating in the department, thus generating outbreaks and isolated cases. In the domestic cycle, the interaction with primary vectors such as *R. prolixus* has been related to climatic factors such as precipitation (26, 27), finding that its frequency in homes increases in times of low rainfall and decreases in the months of higher precipitation (Figure 5). Likewise, the continuous contact of humans mainly in rural areas with reservoirs such as *Didelphis marsupialis* favors the interaction with DTUs of the domestic cycle and could explain the presence of some acute and chronic cases. These climatic conditions could favor the intrusion of secondary vectors in the houses, favoring the appearance of oral outbreaks and isolated acute cases such as those observed by *P. geniculatus* in which sylvatic cycle DTUs involved in the outbreaks are observed, and that typically are related to greater clinical severity and higher parasitemia in both human and animal models.

On the other hand, economic activities developed in the department such as oil exploitation or extensive crops favor human contact with wild transmission cycles, to the point of generating outbreaks of oral transmission, as occurred in the municipality of Paz de Ariporo in 2014, where the responsible DTU was TcI of sylvatic origin through the close contact of wild reservoirs (21). Recently, Casanare has ventured into the agro-industrial cultivation of oil palm (*Elaeis guineensis*) as a potential economic activity in the region, which also favors the invasion of humans in wild cycles and contact with reservoirs and triatomines present in the palms. These economic activities of great importance for the development of the department explain why in our study, the highest frequency of cases occurred in males in people of productive age. Ecologically, the department has a wide distribution of native *Attalea butyracea* palms in which an abundant presence of triatomines has been found, mainly *R. prolixus* (24), challenging the control of the disease.

It is mainly those accidental events (intrusions of vectors and reservoirs in homes and/or human intrusions in sylvatic environments), which are responsible for the appearance of acute cases and outbreaks; coupled with climatic conditions that favor interactions between transmission cycles and the region's ecological conditions, which make Casanare an endemic department for acute Chagas disease. These findings suggest the importance of designing strategies to control triatomine intrusion into homes and strengthening preventive measures in the community and the capacities of medical and paramedical personnel from health institutions, especially during the first quarter of the year.

## Conclusions

Casanare, an endemic department for CD, not only has the highest prevalence of the disease in the country but has also reported the highest number of cases in the acute phase in the period 2012–2020, mainly affecting young adult men in the populated centers. The clinical presentation of the disease is polysymptomatic, almost always accompanied by a prolonged febrile syndrome, with a good prognosis when a timely diagnosis is made. It is necessary to strengthen the importance of immediate diagnosis and treatment of acute CD cases and long-term follow-up of patients. Considering the epidemiological behavior of the disease, it is necessary to establish individual and collective intersectoral public health policies adapted to local settings to achieve the timely detection of acute cases and thus prevent the chronic phase and possible complications.

## Data Availability Statement

The data analyzed in this study is subject to the following licenses/restrictions: the data sets generated analyzed during the current study are not publicly available because they contain sensitive data from the analyzed medical records, however, they are available from the corresponding author upon reasonable request. The information related to the manual collection of vectors is not available for publication, considering that it is part of the community surveillance database of the public health surveillance system of the Health secretariat of Casanare, this information can be requested directly in this institution. Information related to DTUs in Casanare is included in Supplementary Table 2. Data related to IDEAM are in the public domain and can be found at http://atlas.ideam.gov.co/visorAtlasClimatologico.html. Requests to access these datasets should be directed to juand.ramirez@urosario.edu.co.

## Ethics Statement

The studies involving human participants were reviewed and approved by the ethics committee of Universidad del Rosario Act number 426 from July 30th, 2020. Written informed consent for participation was not required for this study in accordance with the national legislation and the institutional requirements.

## Author Contributions

CR-A and AP-G: obtaining information, analysis, writing, and revision and editing of the manuscript. MO: analysis of results and review of methodology of the manuscript. FT-T: information gathering and technical review of the manuscript. LZ-D: technical review of the manuscript. CH: analysis of results DTU Casanare and review of manuscript writing. JR: thematic and methodological review of the manuscript. All authors read and approved the final manuscript.

## Conflict of Interest

The authors declare that the research was conducted in the absence of any commercial or financial relationships that could be construed as a potential conflict of interest.

## Publisher's Note

All claims expressed in this article are solely those of the authors and do not necessarily represent those of their affiliated organizations, or those of the publisher, the editors and the reviewers. Any product that may be evaluated in this article, or claim that may be made by its manufacturer, is not guaranteed or endorsed by the publisher.

## Abbreviations

IDEAM: Instituto de Hidrología, Meteorología y Estudios Ambientales
SD: Standard deviation
DTU: Discrete Typing Units
*T. cruzi*: *Trypanosoma cruzi*
INS: National Institute of Health
*R. prolixus*: *Rhodnius prolixus*
SIVIGILA: Public Health Surveillance System
UPGD: Primary Data Generating Units
IPS: Health Service Provider Institutions
DANE: National Administrative Department of Statistics
*T. maculata*: *Triatoma maculata*
*P. geniculatus*: *Panstrongylus geniculatus*
*T. dimidiata*: *Triatoma dimidiata*
PCR: Polymerase Chain Reaction
qPCR: quantitative Polymerase Chain Reaction
ELISA: Enzyme Immunoassay
IFAT: Immunofluorescence antibody test.
