# Supplementary material for: Clinical and Epidemiological Characterization of Acute Chagas Disease in Casanare, Eastern Colombia, 2012–2020
Source: Front Med (Lausanne). 2021 Jul 23;8:681635. doi: 10.3389/fmed.2021.681635 (PMC8343227; doi:10.3389/fmed.2021.681635)
Supplement: Supplementary file 1 [file Table_1.DOCX]

Supplementary Table 1. Variables usadas en el estudio.

|  | **Denomination** | **Definition** |
| --- | --- | --- |
| **SOCIODEMOGRAPHIC VARIABLES** | Fecha de Notificación | Fecha de notificación del caso agudo. |
|  | Semana de Notificación | Semana de notificación del caso agudo. |
|  | Año | Año de presentación del caso agudo, dato obtenido de la primera historia clínica. |
|  | Edad | Edad en años, meses o días, que corresponde a la edad que tenía el usuario, en la primera consulta que corresponde al caso agudo, tomada de la primera historia clínica. |
|  | Unidad de Medida de Edad | Unidad de medida de la edad, tomada de la primera historia clínica. |
|  | Nacionalidad | Nacional o extranjero, tomado de la primera historia clínica. |
|  | Sexo | Sexo del paciente que presenta el caso agudo, dato obtenido de la primera historia clínica. |
|  | Municipio de procedencia | Municipio de procedencia del caso agudo. |
|  | Departamento de Residencia | Departamento de residencia del paciente con caso agudo de Chagas, reportado en la primera historia clínica o investigación de campo. |
|  | Municipio de Residencia | Municipio de residencia del paciente con caso agudo de Chagas, reportado en la primera historia clínica o investigación de campo. |
|  | Area | Área de procedencia del caso, definida como zona urbana, rural o centro poblado que corresponde a la primera consulta, tomado de la primera historia clínica. |
|  | Ocupación | Ocupación del paciente reportado en la primera historia clínica. |
|  | Tipo de Régimen en Salud | Tipo de régimen en subsidiado-contributivo, registrado en la primera historia clínica |
|  | EAPB | Nombre de la Empresa Administradora de Planes de Beneficios que presta la atención al paciente, dato obtenido de la primera historia clínica. |
|  | Pertenencia Étnica | Grupo étnico al que pertenece el paciente con caso agudo de Chagas, dato obtenido en la primera consulta. |
|  | Grupo Poblacional | Paciente con alguno de lo grupos poblacionales descritos. Si no pertenece a ninguno se coloca otros |
|  | Semanas de Gestación | Semanas de gestación de la paciente gestante con caso agudo de Chagas reportadas en la primera consulta. |
| **CLINICAL VARIABLES** | Fecha de Consulta | Fecha de consulta por parte del usuario en un centro médico, del caso agudo de Chagas, dato obtenido de la primera historia clínica. |
|  | Número de consultas previas a consulta diagnóstica | Número de consultas previas por los mismos síntomas a IPS y otros lugares. |
|  | Fecha Inicio de Síntomas | Fecha aproximada de inicio de síntomas del caso agudo de Chagas, reportado en la primera consulta. |
|  | Hospitalizado | Necesidad de hospitalización por Chagas agudo, dato obtenido en la primera historia clínica o el control, según la evolución que haya tenido el evento. |
|  | Fecha de Hospitalización | Fecha de hospitalización por Chagas agudo, dato obtenido en la primera historia clínica o en el control. |
|  | Días entre de inicio de síntomas y fecha de consulta | Difrencia obtenida entre la fehca de la consulta y la fecha del inicio de los síntomas |
|  | Días de hospitalización | Diferencia entre la fecha de egreso y la fecha de hospitalización |
|  | Condición Final | Condición final (vivo o muerto) del paciente que fue clasificado como caso agudo, y que se presente durante este periodo. Dato obtenido de la primera historia clínica o de cualquiera de los controles realizados durante este periodo. |
|  | Fecha de Defunción | Si la condición final es "muerto", se debe registrar la fecha de la defunción de la persona clasificada como caso agudo. Dato obtenido de la historia clínica que lo registre. |
|  | Días entre la fecha de inicio de síntomas y la fecha de la muerte | Diferencia entre la fecha de inicio de síntomas y la fecha de muerte |
|  | Necropsia | Conclusión hallazgos de necropsia, del caso agudo de Chagas agudo fallecido |
|  | Hallazgo Miocarditis | Miocarditis presente, reportada en la necropsia |
|  | Hallazgo Encefalitis | Encefalitis presente, reportada en la necropsia |
|  | Hallazgo Amastigotes | Presencia de amastigotes, reportados en la necropsia |
|  | Fecha Egreso | Si la condición final es "vivo" y estuvo hospitalizado, se debe registrar la fecha de egreso de la hospitalización de la persona clasificada como caso agudo, Dato obtenido de la historia clínica que lo registre. |
|  | Fiebre | Síntoma y/o signo registrado en la anamnesis y/o examen físico de la persona con caso agudo de Chagas, dato obtenido de la primera historia clínica. |
|  | Medición Temperatura | Medición de la temperatura mas alta, referida por el paciente o medida en cualquier momento de la atención médica |
|  | Días de fiebre | Tiempo medido en días, desde que inició la fiebre, dato obtenido de la primera historia clínica |
|  | Cefalea | Síntoma referido por el paciente, registrado en la primera historia clínica. |
|  | Mialgias | Síntoma referido por el paciente, registrado en la primera historia clínica. |
|  | Artralgias | Síntoma referido por el paciente, registrado en la primera historia clínica. |
|  | Nauseas | Síntoma referido por el paciente, registrado en la primera historia clínica. |
|  | Diarrea | Síntoma referido por el paciente, registrado en la primera historia clínica. |
|  | Vomito | Síntoma referido por el paciente, registrado en la primera historia clínica. |
|  | Epistaxis | Síntoma o signo registrado registrado en la primera historia clínica. |
|  | Dolor Abdominal | Síntoma referido por el paciente, registrado en la primera historia clínica. |
|  | Dolor Retroocular | Síntoma referido por el paciente, registrado en la primera historia clínica. |
|  | Disnea | síntoma referido por el paciente, registrado en la primera historia clínica |
|  | Edema Facial | Síntoma y/o signo registrado en la primera historia clínica |
|  | Edema Periorbitario | Síntoma y/o signo registrado en la primera historia clínica |
|  | Edema Miembros Inferioes | Síntoma y/o Signo registrado en la primera historia clínica |
|  | Hepatomegalia | Signo registrado en la primera historia clínica |
|  | Esplenomegalia | Signo registrado en la primera historia clínica |
|  | Hepatoesplenomegalia | Signo registrado en la primera historia clínica |
|  | Adenopatías | Síntoma y/o signo registrado en la primera historia clínica |
|  | Signo de Romaña | Síntoma y/o signo registrado en la primera historia clínica |
|  | Chagoma | Síntoma y/o signo registrado en la primera historia clínica |
|  | Disfagia | Síntoma referido por el paciente, registrado en la primera historia clínica. |
|  | Dolor Torácico | Síntoma referido por el paciente, registrado en la primera historia clínica. |
|  | Palpitaciones | Síntoma referido por el paciente, registrado en la primera historia clínica. |
|  | Bradicardia | Signo registrado en la primera historia clínica. |
|  | Taquicardia | Signo registrado en la primera historia clínica. |
|  | Taquipnea | Signo registrado en la primera historia clínica. |
|  | Ictericia | Síntoma y/o signo registrado en la primera historia clínica |
|  | Coluria | Síntoma y/o signo registrado en la primera historia clínica |
|  | Otros Síntomas | Otros síntomas referidos por el paciente, registrado en la primera historia clínica. |
|  | Diagnóstico Diferencial Inicial | Impresión diagnóstica o diagnósticos previos realizados antes de la confirmación de la enfermedad de Chagas agudo, registrados en la historia clínica |
|  | Arritmia Cardiaca | Diagnóstico registrado en la primera historia clínica o evoluciones como complicación de la enfermedad de Chagas agudo. |
|  | Fibrilación auricular o flutter auricular | Diagnóstico registrado en la primera historia clínica o evoluciones como complicación de la enfermedad de Chagas agudo. |
|  | Derrame Pericárdico | Diagnóstico registrado en la primera historia clínica o evoluciones como complicación de la enfermedad de Chagas agudo. |
|  | Taponamiento Cardiaco | Diagnóstico registrado en la primera historia clínica o evoluciones como complicación de la enfermedad de Chagas agudo. |
|  | Falla Cardiaca | FALLA CARDIACA O INSUFICIENCIA CARDIACA. Diagnóstico registrado en la primera historia clínica o evoluciones como complicación de la enfermedad de Chagas agudo. |
|  | Pericarditis | Diagnóstico registrado en la primera historia clínica o evoluciones como complicación de la enfermedad de Chagas agudo. |
|  | Miocarditis | Diagnóstico registrado en la primera historia clínica o evoluciones como complicación de la enfermedad de Chagas agudo. |
|  | Meningoencefalitis | Diagnóstico registrado en la primera historia clínica o evoluciones como complicación de la enfermedad de Chagas agudo. |
|  | Gota Gruesa | Prueba parasitológica directa en busca del parásito en fase aguda, registrado en cualquier consulta como diagnóstico de la enfermedad. |
|  | Micrométodo | Prueba parasitológica directa en busca del parásito en fase aguda, registrado en cualquier consulta como diagnóstico de la enfermedad. |
|  | Frotis o extendido de sangre periférica | Prueba parasitológica directa en busca del parásito en fase aguda, registrado en cualquier consulta como diagnóstico de la enfermedad. |
|  | Examen directo de sangre fresca | Prueba parasitológica directa en busca del parásito en fase aguda, registrado en cualquier consulta como diagnóstico de la enfermedad |
|  | Micro hematocrito | Prueba parasitológica directa en busca del parásito en fase aguda, registrado en cualquier consulta como diagnóstico de la enfermedad. |
|  | Strout | Prueba parasitológica directa en busca del parásito en fase aguda, registrado en cualquier consulta como diagnóstico de la enfermedad. |
|  | ELISA Ag Totales (convencional) | Prueba serológica en busca de IgG anti *T.cruzi*, realizados en los laboratorios de referencia. |
|  | ELISA Ag Recombinantes/Peptidos sintéticos (no convencional) | Prueba serológica en busca de IgG anti *T.cruzi*, realizados en los laboratorios de referencia. |
|  | Inmunofluorescencia Indirecta IgG *T. Cruzi* | Prueba serológica en busca de IgG anti *T.cruzi*, realizados en los laboratorios de referencia. |
|  | Inmunoblot | Prueba serológica en busca de IgG anti *T.cruzi*, realizados en los laboratorios de referencia. |
|  | Proteína C Reactiva | Prueba molecular en busca de IgG anti *T.cruzi*, realizados en los laboratorios de referencia. |
|  | Hematoaglutinación Indirecta | Prueba serológica en busca de IgG anti *T.cruzi*, realizados en los laboratorios de referencia. |
|  | Radiografía de Tórax | Resultado de imagen diagnóstica en paciente confirmado con enfermedad de Chagas agudo. |
|  | Radiografía de Tórax anormal | Reporte de radiografía de torax con alguna anormalidad. |
|  | Cardiomegalia | Cardiomegalia descrita en el reporte de la radiografía de torax. Dato obtenido de la historia clínica |
|  | Derrame pleural | Derrame pleural descrito en el reporte de la radiografía de torax. Dato obtenido de la historia clínica |
|  | Electrocardiograma | Resultado de imagen diagnóstica en paciente confirmado con enfermedad de Chagas agudo. |
|  | Electrocardiograma anormal | Reporte del electrocardiograma descrito en la historia clínica anormal |
|  | Taquicardia sinusal | Taquicardia sinusal descrito en el electrocardiorama y reportado en la historia clínica |
|  | Bradicardia sinusal | Bradicardia sinusal descrito en el electrocardiorama y reportado en la historia clínica |
|  | Bloqueo Auriculoventricular | Bloqueo Auriculoventricular descrito en el electrocardiorama y reportado en la historia clínica |
|  | Bloqueo de rama derecha | Bloqueo de rama derecha descrito en el electrocardiorama y reportado en la historia clínica |
|  | Bloqueo de rama izquierda | Bloqueo de rama izquierda descrito en el electrocardiorama y reportado en la historia clínica |
|  | Extrasistoles ventriculares | Extrasistoles ventriculares descrito en el electrocardiorama y reportado en la historia clínica |
|  | Trastorno inespecífico de la repolarización | Trastorno inespecífico de la repolarización descrito en el electrocardiorama y reportado en la historia clínica |
|  | Fibrilación auricular | Fibrilación auricular descrito en el electrocardiorama y reportado en la historia clínica |
|  | Supradesnivel de ST | Supradesnivel de ST descrito en el electrocardiorama y reportado en la historia clínica |
|  | Inversión Onda T | Inversión Onda T descrito en el electrocardiorama y reportado en la historia clínica |
|  | Ecocardiograma | Resultado de imagen diagnóstica en paciente confirmado con enfermedad de Chagas agudo. |
|  | Ecocardiograma anormal | Reporte del ecocardiograma descrito en la historia clínica con alguna anormalidad |
|  | Derrame pericardico | Derrame pericardico reportado en el ecocardiograma y descrito en la historia clínica |
|  | Taponamiento pericardico | Taponamiento cardiaco reportado en el ecocardiograma y descrito en la historia clínica |
|  | Ecografía abdominal | Resultado de imagen diagnóstica en paciente confirmado con enfermedad de Chagas agudo. |
|  | Hepatomegalia | Hepatomegalia reportada en la ecografía abdominal, dato obtenio de la historia clínica |
|  | Esplenomegalia | Esplenomegalia reportada en la ecografía abdominal, dato obtenio de la historia clínica |
|  | Holter | Resultado de imagen diagnóstica en paciente confirmado con enfermedad de Chagas agudo. |
|  | Recuento leucocitario | Resultado de hemograma/cuado hemático en paciente confirmado con enfermedad de Chagas agudo. |
|  | Línea celular predominante en el cuadro hemático | Resultado de hemograma/cuadro hemático en paciente confirmado con enfermedad de Chagas agudo. |
|  | Hemoglobina | Resultado de hemograma/cuado hemático en paciente confirmado con enfermedad de Chagas agudo. |
|  | Hematocrito | Resultado de hemograma/cuadro hemático en paciente confirmado con enfermedad de Chagas agudo. |
|  | Plaquetas | Resultado de hemograma/cuado hemático en paciente confirmado con enfermedad de Chagas agudo. |
|  | Macroplaquetas | Resultado de hemograma/cuadro hemático o extendido de sangre periférico en paciente confirmado con enfermedad de Chagas agudo. |
|  | Transaminasa glutámico oxalacética TGO | Resultado de TGO en paciente confirmado con enfermedad de Chagas agudo. |
|  | Transaminasa glutámica pirúvica TGP | Resultado de TGP en paciente confirmado con enfermedad de Chagas agudo. |
|  | Creatinina | Resultado de creatinina en paciente confirmado con enfermedad de Chagas agudo. |
|  | Nitrógeno Ureico BUN | Resultado de nitrógeno ureico (BUN) en paciente confirmado con enfermedad de Chagas agudo. |
|  | Glucosa | Resultado de glucosa en sangre en paciente confirmado con enfermedad de Chagas agudo. |
|  | Tiempo de trombina | Resultado de PT en paciente confirmado con enfermedad de Chagas agudo. |
|  | Tiemp de tromboplastina | Resultado de PTT en paciente confirmado con enfermedad de Chagas agudo. |
|  | Tratamiento Etiológico | Tratamiento antiparasitario instaurado posterior al diagnóstico, dato obtenido de la historia clínica. |
|  | Tipo de Medicamento | Tipo de medicamento utilizado para el tratamiento etiológico, dato obtenido de la historia clínica. |
|  | Complicación del tratamiento | Complicación secundaria al medicamento recibido Benzonidazol o Nifurtimox, dato encontrado en los controles realizados posterior al diagnóstico. |
|  | Tipo complicación del tratamiento | Tipo de complicación secundaria al medicamento recibido Benzonidazol o Nifurtimox, dato encontrado en los controles realizados posteriores al diagnóstico. |
|  | Cambio de medicamento por complicación | Cambio de medicamento antiparasitario secundario a la complicación por el tratamiento antiparasitario inicial. Dato obtenido de controles de historia clínica. |
| **EPIDEMIOLOGICAL VARIABLES** | Antecedentes de picadura del vector | Antecedente referido por el paciente o el familiar en historia clínica o investigación de campo picadura del vector |
|  | Avistamiento de vector | Avistamiento del vector referido por paciente o familiar, intra, peri o extradomiciliario. |
|  | Avistamiento de zarigüellas | Avistamiento de zarigüellas referido por paciente o familiar, intra, peri o extradomiciliario. |
|  | Brote | Caso de Enfermedad de Chagas agudo confirmado en el marco de un brote |
|  | Problable Vía de Transmisión | Vía de transmisión establecido en la historia clínica o en el estudio del caso. |
|  | Clasificación de caso | Clasificación del caso, que con síntomas y signos de Chagas agudo, y según el tipo de caso, fue clasificado como agudo. Dato obtenido de la consulta en la que se haya hecho el diagnóstico y la clasificación. |
|  | Tipo de Caso | Tipo de caso para Chagas agudo notificado, dato obtenido en la primera consulta o en el control. |
|  | Reactivación | Paciente con antecedente de patologías con compromiso de inmunidad celular (VIH, inmunosuprimidos por cualquier causa), que presentan caso agudo de Chagas, notificado en la primera consulta. |
