# Supplementary material for: Clinical and Epidemiological Characterization of Acute Chagas Disease in Casanare, Eastern Colombia, 2012–2020
Source: Front Med (Lausanne). 2021 Jul 23;8:681635. doi: 10.3389/fmed.2021.681635 (PMC8343227; doi:10.3389/fmed.2021.681635)
Supplement: Supplementary file 2 [file Table_2.DOCX]

Supplementary Table 2. Publications that describe the DTUs identified in Casanare and Colombia 2011-2020.

|  | **Ref** | **Title** | **Year** |
| --- | --- | --- | --- |
| 1 | (1) | Temporal Variation of the Presence of Rhodnius prolixus (Hemiptera: Reduviidae) Into Rural Dwellings in the Department of Casanare, Eastern Colombia. | 2020 |
| 2 | (2) | *Trypanosoma cruzi* infection, discrete typing units and feeding sources among *Psammolestes arthuri* (Reduviidae: Triatominae) collected in eastern Colombia | 2019 |
| 3 | (3) | Generalist host species drive *Trypanosoma cruzi* vector infection in oil palm plantations in the Orinoco region, Colombia | 2019 |
| 4 | (4) | Importation of Hybrid Human-Associated *Trypanosoma cruzi* Strains of Southern South American Origin, Colombia | 2016 |
| 5 | (5) | High-Resolution Molecular Typing of *Trypanosoma cruzi* in 2 Large Outbreaks of Acute Chagas Disease in Colombia | 2016 |
| 6 | (6) | Risks associated with dispersive nocturnal flights of sylvatic Triatominae to artificial lights in a model house in the northeastern plains of Colombia | 2015 |
| 7 | (7) | Follow-up of an Asymptomatic Chagas Disease Population of Children after Treatment with Nifurtimox (Lampit) in a Sylvatic Endemic Transmission Area of Colombia | 2015 |
| 8 | (8) | Retrospective distribution of *Trypanosoma cruzi* I genotypes in Colombia. | 2015 |
| 9 | (9) | Specific primers design based on the superoxide dismutase b gene for *Trypanosoma cruzi* as a screening tool: Validation method using strains from Colombia classified according to their discrete typing unit. | 2014 |
| 10 | (10) | Trypanosome species in neo-tropical bats: biological, evolutionary and epidemiological implications | 2014 |
| 11 | (11) | Retrospective molecular integrated epidemiology of Chagas disease in Colombia. | 2013 |
| 12 | (12) | Phylogenetic reconstruction based on Cytochrome b (Cytb) gene sequences reveals distinct genotypes within Colombian *Trypanosoma cruzi* I populations. | 2011 |

**References**

1. Rincón-Galvis HJ, Urbano P, Hernández C, Ramírez JD. Temporal Variation of the Presence of Rhodnius prolixus (Hemiptera: Reduviidae) Into Rural Dwellings in the Department of Casanare, Eastern Colombia. Florin D, editor. J Med Entomol [Internet]. 2020 Jan 9;57(1):173–80. Available from: https://academic.oup.com/jme/article/57/1/173/5574699

2. Velásquez-Ortiz N, Hernández C, Herrera G, Cruz-Saavedra L, Higuera A, Arias-Giraldo LM, et al. Trypanosoma cruzi infection, discrete typing units and feeding sources among Psammolestes arthuri (Reduviidae: Triatominae) collected in eastern Colombia. Parasit Vectors [Internet]. 2019 Dec 8;12(1):157. Available from: https://parasitesandvectors.biomedcentral.com/articles/10.1186/s13071-019-3422-y

3. Erazo D, Gottdenker NL, González C, Guhl F, Cuellar M, Kieran TJ, et al. Generalist host species drive Trypanosoma cruzi vector infection in oil palm plantations in the Orinoco region, Colombia. Parasit Vectors [Internet]. 2019 Dec 28;12(1):274. Available from: https://parasitesandvectors.biomedcentral.com/articles/10.1186/s13071-019-3519-3

4. Messenger LA, Ramirez JD, Llewellyn MS, Guhl F, Miles MA. Importation of Hybrid Human-Associated Trypanosoma cruzi Strains of Southern South American Origin, Colombia. Emerg Infect Dis [Internet]. 2016 Aug;22(8):1452–5. Available from: http://wwwnc.cdc.gov/eid/article/22/8/15-0786_article.htm

5. Hernández C, Vera MJ, Cucunubá Z, Flórez C, Cantillo O, Buitrago LS, et al. High-Resolution Molecular Typing of Trypanosoma cruzi in 2 Large Outbreaks of Acute Chagas Disease in Colombia. J Infect Dis [Internet]. 2016 Oct 15;214(8):1252–5. Available from: https://academic.oup.com/jid/article-lookup/doi/10.1093/infdis/jiw360

6. Jácome-Pinilla D, Hincapie-Peñaloza E, Ortiz MI, Ramírez JD, Guhl F, Molina J. Risks associated with dispersive nocturnal flights of sylvatic Triatominae to artificial lights in a model house in the northeastern plains of Colombia. Parasit Vectors [Internet]. 2015 Dec 19;8(1):600. Available from: http://www.parasitesandvectors.com/content/8/1/600

7. Bianchi F, Cucunubá Z, Guhl F, González NL, Freilij H, Nicholls RS, et al. Follow-up of an Asymptomatic Chagas Disease Population of Children after Treatment with Nifurtimox (Lampit) in a Sylvatic Endemic Transmission Area of Colombia. Franco-Paredes C, editor. PLoS Negl Trop Dis [Internet]. 2015 Feb 27;9(2):e0003465. Available from: https://dx.plos.org/10.1371/journal.pntd.0003465

8. León CM, Hernández C, Montilla M, Ramírez JD. Retrospective distribution of Trypanosoma cruzi I genotypes in Colombia. Mem Inst Oswaldo Cruz [Internet]. 2015 Apr 28;110(3):387–93. Available from: http://www.scielo.br/scielo.php?script=sci_arttext&pid=S0074-02762015000300387&lng=en&tlng=en

9. Olmo F, Escobedo-Orteg J, Palma P, Sánchez-Moreno M, Mejía-Jaramillo A, Triana O, et al. Specific primers design based on the superoxide dismutase b gene for Trypanosoma cruzi as a screening tool: Validation method using strains from Colombia classified according to their discrete typing unit. Asian Pac J Trop Med [Internet]. 2014 Nov;7(11):854–9. Available from: http://linkinghub.elsevier.com/retrieve/pii/S1995764514601498

10. Ramírez JD, Tapia-Calle G, Muñoz-Cruz G, Poveda C, Rendón LM, Hincapié E, et al. Trypanosome species in neo-tropical bats: Biological, evolutionary and epidemiological implications. Infect Genet Evol [Internet]. 2014 Mar;22:250–6. Available from: https://linkinghub.elsevier.com/retrieve/pii/S1567134813002475

11. Guhl F, Ramírez JD. Retrospective molecular integrated epidemiology of Chagas disease in Colombia. Infect Genet Evol [Internet]. 2013 Dec;20:148–54. Available from: https://linkinghub.elsevier.com/retrieve/pii/S156713481300333X

12. Ramírez JD, Duque MC, Guhl F. Phylogenetic reconstruction based on Cytochrome b (Cytb) gene sequences reveals distinct genotypes within Colombian Trypanosoma cruzi I populations. Acta Trop [Internet]. 2011 Jul;119(1):61–5. Available from: https://linkinghub.elsevier.com/retrieve/pii/S0001706X11000799
