# Supplementary material for: Clinical and Epidemiological Characterization of Acute Chagas Disease in Casanare, Eastern Colombia, 2012–2020
Source: Front Med (Lausanne). 2021 Jul 23;8:681635. doi: 10.3389/fmed.2021.681635 (PMC8343227; doi:10.3389/fmed.2021.681635)
Supplement: Supplementary file 3 [file Table_3.DOCX]

Supplementary Table 3. Demographic characteristics, acute Chagas cases, Casanare 2012-2020.

| **Categorical variable** | **No. cases** | |
| --- | --- | --- |
|  | **Frecuency (n = 103)** | **%** |
| **Sex** |  |  |
| Male | 72 | 69.9 |
| Female | 31 | 30.1 |
| **Area of origin** |  |  |
| Urban | 4 | 3.9 |
| Rural | 99 | 96.1 |
| **Membership type SGSSS** |  |  |
| Subsidized | 43 | 41.7 |
| Contributory | 59 | 57.3 |
| Not affiliated | 1 | 1.0 |
| **Age group** |  |  |
| 0 a 4 | 6 | 5.8 |
| 5 a 14 | 9 | 8.7 |
| 15 a 29 | 29 | 28.2 |
| 30 a 44 | 35 | 34.0 |
| 45 a 64 | 22 | 21.4 |
| ≥ 65 | 2 | 1.9 |
