# Supplementary material for: Clinical and Epidemiological Characterization of Acute Chagas Disease in Casanare, Eastern Colombia, 2012–2020
Source: Front Med (Lausanne). 2021 Jul 23;8:681635. doi: 10.3389/fmed.2021.681635 (PMC8343227; doi:10.3389/fmed.2021.681635)
Supplement: Supplementary file 4 [file Table_4.DOCX]

Supplementary Table 4. Laboratory tests used in the diagnosis of acute Chagas, Casanare 2012-2020.

| **Diagnostic test** | **Test Results** | **Frecuency** | **%** |
| --- | --- | --- | --- |
| Thick Smear | Positive | 58 | 56.3 |
|  | Negative | 36 | 35.0 |
| Micromethod | Positive | 16 | 15.5 |
|  | Negative | 18 | 17.5 |
| Strout | Positive | 10 | 9.7 |
|  | Negative | 31 | 30.1 |
| Thick Blood Smears | Positive | 9 | 8.7 |
|  | Negative | 35 | 34.0 |
| Fresh Blood Smear | Positive | 5 | 4.9 |
|  | Negative | 36 | 35.0 |
| Microhematocrit | Positive | 2 | 1.9 |
|  | Negative | 32 | 31.1 |
| PCR* | Positive | 24 | 23.3 |
| ELISA | Positive | 74 | 71.8 |
|  | Negative | 4 | 3.9 |
| IFAT | Reactive | 59 | 57.3 |
|  | Non Reactive | 1 | 1 |

* The PCRs were performed following the protocols reported and validated by (1,2)

**References**

1. Hernández C, Cucunubá Z, Flórez C, Olivera M, Valencia C, Zambrano P, et al. Molecular Diagnosis of Chagas Disease in Colombia: Parasitic Loads and Discrete Typing Units in Patients from Acute and Chronic Phases. Debrabant A, editor. PLoS Negl Trop Dis [Internet]. 2016 Sep 20;10(9):e0004997. Available from: https://dx.plos.org/10.1371/journal.pntd.0004997

2. Hernández C, Vera MJ, Cucunubá Z, Flórez C, Cantillo O, Buitrago LS, et al. High-Resolution Molecular Typing of Trypanosoma cruzi in 2 Large Outbreaks of Acute Chagas Disease in Colombia. J Infect Dis [Internet]. 2016 Oct 15;214(8):1252–5. Available from: https://academic.oup.com/jid/article-lookup/doi/10.1093/infdis/jiw360
